# A novel candidate hepatitis C virus genotype 4 subtype identified by next generation sequencing full-genome characterization in a patient from Saudi Arabia

**DOI:** 10.3389/fmicb.2023.1285367

**Published:** 2023-11-02

**Authors:** Mariantonietta Di Stefano, Mona H. Ismail, Thomas Leitner, Giuseppina Faleo, Marwan Jabr Alwazzeh, Jean Lutamyo Mbisa, Josè Ramon Fiore, Teresa Antonia Santantonio

**Affiliations:** ^1^Section of Infectious Diseases, Department of Clinical and Surgical Sciences, University of Foggia, Foggia, Italy; ^2^Division of Gastroenterology, King Fahd Hospital of the University, Al-Khobar, Saudi Arabia; ^3^College of Medicine, Imam Abdulrahman Bin Faisal University, Dammam, Saudi Arabia; ^4^Theoretical Biology and Biophysics Group, Los Alamos National Laboratory, Los Alamos, NM, United States; ^5^Infectious Disease Division, King Fahd Hospital of the University, Al-Khobar, Saudi Arabia; ^6^Antiviral Unit, Blood Safety, Hepatitis, Sexually Transmitted Infections, and HIV (BSHSH) Service, UK Health Security Agency, London, United Kingdom

**Keywords:** Hepatitis C virus (HCV), genotypes, subtypes, next generation sequence, Saudi Arabia

## Abstract

**Background and aim:**

Hepatitis C virus (HCV) infection is a major global public health concern, being a leading cause of chronic liver diseases such as chronic hepatitis, cirrhosis, and hepatocellular carcinoma. The virus is classified into 8 genotypes and 93 subtypes, each displaying distinct geographic distributions. Genotype 4 is the most predominant in the Middle East and Eastern Mediterranean and is associated with high rates of hepatitis C infection worldwide. This study used next-generation sequencing to fully characterize the HCV genome and identify a novel subtype within genotype 4 isolated from a 64-year-old Saudi man diagnosed with hepatitis C.

**Methods:**

We analyzed the complete genome of the 141-HCV isolate using whole-genome sequencing.

**Results:**

Our phylogenetic reconstructions, based on the entire genome of HCV-4 strains, revealed that the 141-HCV isolate formed a distinct group within the genotype 4 classification, providing valuable new insights into the variability of HCV.

**Conclusion:**

This discovery of a previously unclassified HCV subtype within genotype 4 sheds light on the ongoing evolution and diversity of the virus. Such knowledge has significant implications for diagnostic and therapeutic approaches, as different subtypes may exhibit varying drug sensitivities and resistance profiles.

## Introduction

Hepatitis C virus (HCV) is a single-stranded, positive polarity RNA virus belonging to the genus Hepacivirus, a member of the Flaviviridae family (Li and Lo, 2015). Despite new, highly efficient antiviral drugs, HCV infection is still a major global public health problem, being a major cause of chronic hepatitis, chronic liver diseases, cirrhosis, and hepatocellular carcinoma (Bertino et al., 2016). The Middle East and Eastern Mediterranean (M and E) region have been reported to have the highest rates of HCV infection in the world, with an incidence of 62.5 per 100,000 person-years and a prevalence of 2.3% among the general population. In 2015, it was estimated that approximately one-fourth of 1.75 million newly HCV- infected persons and one-fifth of 71 million chronically infected individuals in the world resided in M and E countries. The median of the anti-HCV seropositivity rate in this region ranged broadly from 0.3% in Iran to 13.0% in Egypt. In Saudi Arabia, the seroprevalence was estimated between 0.4 and 7.3% of the general population (Abdel-Moneim et al., 2012; Alghamdi et al., 2016).

Based on genetic differences, HCV strains are classified into 8 genotypes (Borgia et al., 2018) and 93 subtypes as of March 2022 (International Committee on Taxonimy of Viruses (ICTV), 2018), which vary in geographical distribution. Studying this aspect is vital to define better HCV epidemiology, evolution, and clinically relevant therapeutic approaches. Recently, we characterized several HCV isolates from Saudi Arabia as belonging to different genotype 4 subtypes: “a” (*n* = 9), “o” (*n* = 2), “d” (*n* = 3), two possible recombinants (consisting of GT 4a/GT 4o/GT4a) and one patient with an unclassifiable GT 4 subtype (Di Stefano et al., 2021).

Here, we report a candidate novel HCV genotype 4 variant identified in one of these cases that showed significant genetic diversity and evolution from other characterized HCV genotype 4 isolates. The full-length genome of this novel HCV strain was obtained by using a sequence agnostic whole genome sequence approach, demonstrating that genetically divergent and unknown HCV subtypes are circulating in the human population in Saudi Arabia.

## Materials and methods

### Subject and sample

In this study, we characterized the 141-HCV isolate obtained from the plasma of a 64-year-old heterosexual Saudi man with no risk factors for HCV infection. The patient tested positive for anti- HCV in January 2010 (Architect HCV enzyme immunoassay, Abbott Diagnostic, Chicago, IL, United States). He was later referred to the Hepatology clinic in April 2019 at King Fahd Hospital of the University, Alkhobar, Saudi Arabia. His initial investigation showed that complete blood count, serum aminotransferase levels, bilirubin, albumin, and prothrombin time were within the normal range. HCV RNA in the plasma sample was 6.81 logs IU/mL using the Abbott RealTime HCV assay (Abbott, Rungis, France). However, his initial assessment of liver fibrosis by Fibroscan (502 Touch, Echosens, Paris, France) in May 2019 revealed advanced fibrosis/cirrhosis F3–F4, S0 [Elastography (E) for fibrosis (F):13.2 kPa, Controlled Attenuation Parameter (CAP) for steatosis (S):196 dB/m]. Initial abdominal ultrasound showed features of an irregular liver surface with no splenomegaly or dilated portal vein. Upper endoscopy showed no signs or features of portal hypertension.

For treatment, the patient received a combination of sofosbuvir and daclatasvir in 2019 for 3 months, which resulted in a cure and a successful sustained virological response. Subsequently, a follow-up Fibroscan in 2020 showed F3 and S1 (E: 10.2 kPa, and CAP: 257 dB/m), while in April 2022, showed a significant improvement and regression of liver fibrosis, with F0/S1 (E:4.5 kPa/252 dB/m), indicating a positive response to HCV anti-viral therapy.

An unclassifiable GT4 subtype was initially detected by amplifying and sequencing three different genes (NS3, NS5A, and NS5B) (Di Stefano et al., 2021). For a more comprehensive virus analysis, the HCV strain’s entire genomic sequence (141) was sequenced using sequential capture technology.

### HCV whole genome sequencing

The complete genomic sequence of the HCV isolate was obtained using a previously described state- of-the-art sequence capture methodology. The TAKARA SMARTer Stranded Total RNA-Seq Kit v2 was used to generate libraries from DNAse-digested nucleic acid extracts. Libraries were pooled according to both total mass and HCV-specific fragment frequency. Pools were hybridized to customized HCV-specific biotinylated oligonucleotide probes, designed to cover the diversity of HCV genotypes 1–8, with enriched fragments partitioned onto streptavidin beads and subjected to further cycles of PCR amplification before being sequenced on an Illumina MiSeq instrument. Human reads were removed from the de-multiplexed, adapter-stripped, paired-nend FASTQ datasets by mapping against the hg38 reference. Surviving paired-end reads were mapped to an HCV reference genome set, and the subtypes of each genome match collated to determine the initial subtype composition. In parallel, whole genomes were built with a combination of *de novo* assembly (Manso et al., 2020) to derive a nucleotide frequency table. From this table, a consensus sequence was inferred using a depth threshold of 30 reads per position (Ns were coded at loci with insufficient reads), and positions with mixtures greater than 15% were coded as IUPAC ambiguities.

### Phylogenetic evaluation

The complete genome sequence of the 141-HCV isolate was aligned to 238 HCV reference sequences from the International Committee on Taxonomy of Viruses (ICTV) version 09.03.22, downloaded 2022-10-06, using MAFFT version 7 (Katoh and Standley, 2013). Genotype and subtype were determined by phylogenetic reconstruction using PhyML 3.0 (Guindon et al., 2010) under a GTR + G + I substitution model with NNI + SPR search and aLRT branch support to assess phylogenetic robustness. For assessment of potential recombinant origin, sample 10,002 was also analyzed using PhyloPlace,^1^ which implements the branching index (BI) to assess phylogenetic relatedness in genomic windows of 400 nt moved in 80 nt steps across the genome and a BI threshold of 0.69 (Hraber et al., 2008). Phylogenetic verification was also done with HCV-Glue.^2^

## Results

**Figure 1:**
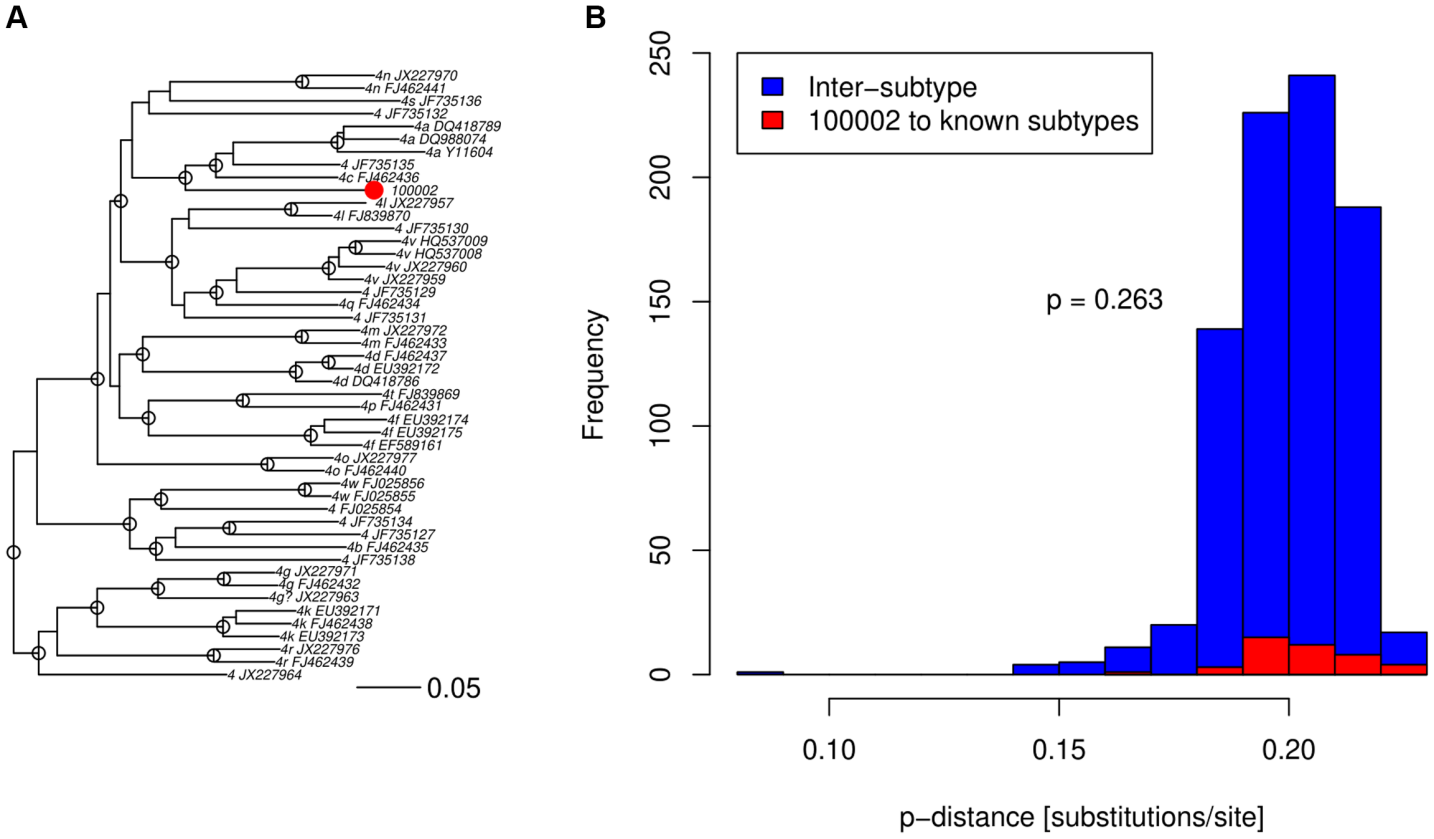
HCV genotype 4 comparisons to the 141-HCV isolate. **(A)** Maximum-likelihood tree comparing the 141-HCV isolate (red dot) to ICTV genotype 4 subtype reference sequences, labeled with subtype and GenBank accession number. Robust phylogenetic support is indicated by an open circle at nodes with aLRT 0.95. **(B)** Pairwise *p*-distances among known subtypes (blue) and between 141-HCV isolate and all known subtypes (red). *p* value indicates true location shift is not equal to 0 between the two distributions using a Wilcoxon rank sum test with continuity correction.

**Figure 2:**
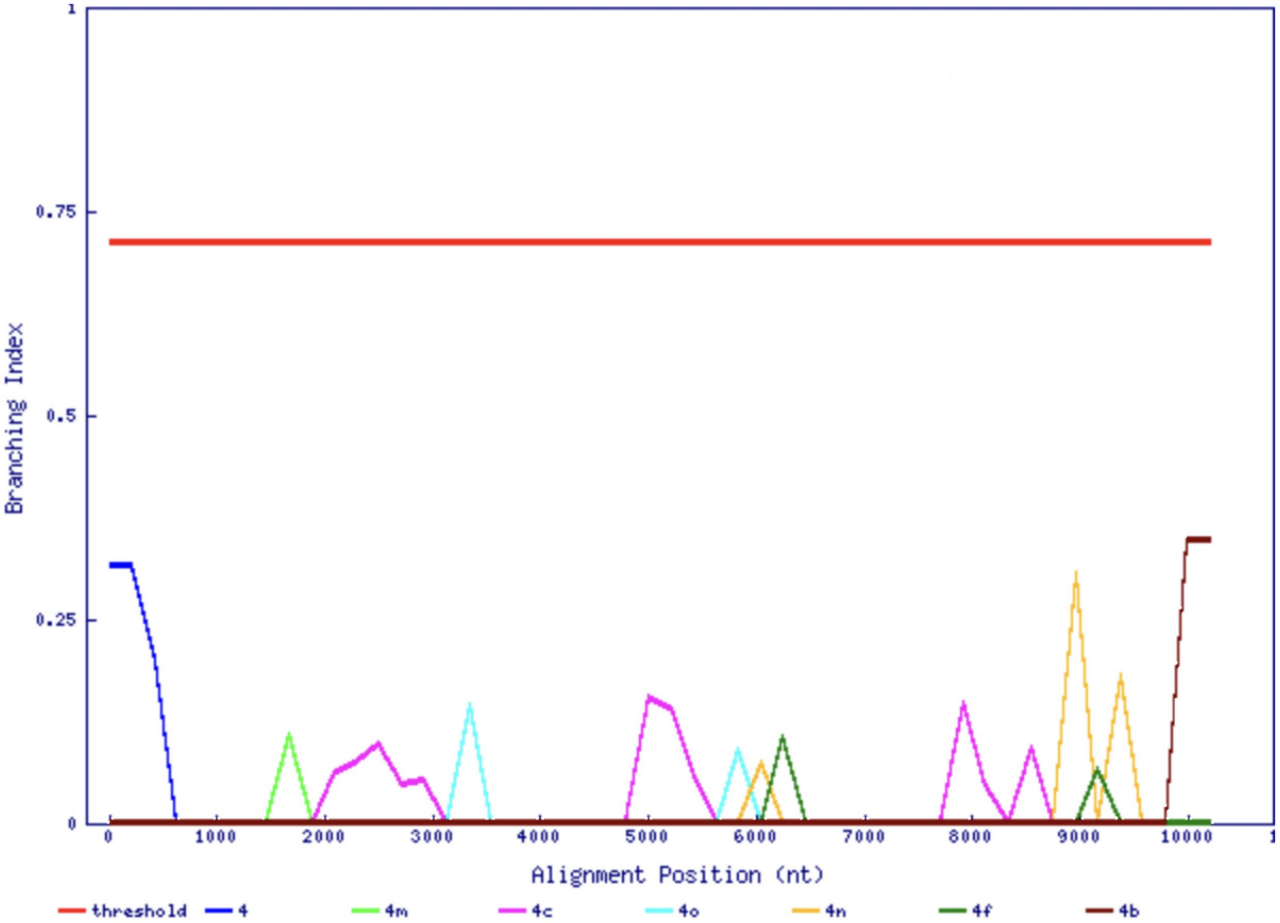
Branching index analysis of the 141-HCV isolate. The red horizontal line indicates significant genotype association. The 141-HCV isolate does not associate with any known genotype across its genome (*x*-axis).

As part of a survey of HCV infection in Saudi Arabia, we previously found an unclassifiable subtype within genotype 4 in an isolate based on the sequencing of NS3, NS5A, and NS5B regions (Di Stefano et al., 2021). In agreement with the previous analysis of the three shorter regions, the complete genome sequence of the 141-HCV isolate did not cluster inside any previously known subtype, nor any reference sequences in the ICTV set that may represent other new subtypes, e.g., JX227964 and JX227963 (Figure 1A). The 141-HCV isolate formed a distinct branch with no reference sequences nearby. Furthermore, the 141-HCV isolate was at a typical genotype 4 inter-subtype distance (Figures 1B, *p*= 0.263, Wilcoxon rank sum test with continuity correction, using 6,483 sequences from 17 genotype 4 subtypes). We investigated the branching index (Wilbe et al., 2003) across the genome to assess the potential recombinant origin. Figure 2 shows that no part of the 141-HCV isolate genome had an origin close enough to any known subtype of genotype 4. All parts of the 141-genome sequence were far below the HCV BI threshold of 0.69, indicating a recombinant origin; thus, this isolate represents a new candidate subtype of HCV genotype 4.

## Discussion

HCV has been shown to have high genetic diversity and is currently classified into 8 genotypes with distinct geographical distributions. The HCV GT 4 is the most dominant in the Middle East and Central and East Africa. In contrast, HCV GT1 is the most common in North and South America, Europe, and Australia, while HCV GT 3 is the most prevalent in the Indian subcontinent. On the other hand, GT 2 is most common in West Africa, GT 5 in South Africa, and GT 6 in Southeast Asia. Finally, the newly discovered genotypes 7 and 8 are dominant in Central Africa and the subcontinent of India, respectively.

Interestingly, increased human migration leads to gradual changes in the distribution of HCV genotype, although the picture of circulating HCV diverging strains is not yet complete. Divergent HCV GT1 has been reported in Germany (Wang et al., 2019) and Cyprus (Oikonomopoulou et al., 2017), while in Canada, several divergent HCV GT 2 were observed, mainly in patients of African origin (Li et al., 2012). However, to classify it as a new HCV subtype, the characterization of three HCV isolates that cluster but are not epidemiologically linked is required (Smith et al., 2014). In the case of HCV genotype 4, four different sub-genotypes (4d, 4r, 4 L, and 4v) were reported in Ethiopia (Hundie et al., 2017). Although subgenotype 5a is the most predominant in South Africa, other subgenotypes and recombinant viruses could be found (Simmonds et al., 2005), and these recombination events could play a significant role in the evolution of RNA viruses. We should also consider that HCV GT 4 epidemiology is changing. It started to expand beyond its strongholds in Africa and the Middle East to several Western countries, especially in Europe, due to changes in demographic structure, immigration, and injection drug use (de Bruijne et al., 2009; Ciccozzi et al., 2012).

Understanding the different genotypes and subtypes of HCV is crucial as they can significantly impact the success of treatment and clinical outcomes of HCV infections (Schinazi et al., 2014; Waldenström et al., 2016). For instance, Interferon-based therapy has shown higher success rates for GT 2 and 3 (Waldenström et al., 2016), while the first- generation HCV protease inhibitors were more effective in GT 1 (Schinazi et al., 2014). Fortunately, second-generation direct acting antivirals have broader coverage across genotypes. However, despite the promise of pangenotypic regimens, they are expensive and often unavailable in low or middle-income countries. As a result, access to these treatments becomes a challenge for many patients in these regions.

These findings demonstrate that through phylogenetic reconstructions using the entire genome of HCV-4 strains, we found that the 141-HCV isolate formed a distinct cluster within the HCV-4 group. This provides valuable new information about the variability of HCV and sheds light on the unique characteristics of this particular strain.

## Data availability statement

The data presented in the study are deposited in the GenBank repository, accession number OR594285.

## Ethics statement

This study was performed according to the Declaration of Helsinki, and ethical approval was obtained from Imam Abdulrahman Bin Faisal University (IRB-2020-01-313, 26 October 2020). The study was conducted in accordance with the local legislation and institutional requirements. The participants provided their written informed consent to participate in this study.

## Author contributions

MD: Conceptualization, Supervision, Writing – original draft, Writing – review & editing. MI: Data curation, Investigation, Writing – review & editing. TL: Formal analysis, Funding acquisition, Investigation, Writing – review & editing. GF: Methodology, Investigation, Writing – original draft. JLM: Formal analysis, Investigation, Writing – review & editing. MT: Investigation, Methodology, Writing – review & editing. JF: Conceptualization, Supervision, Writing – original draft, Writing – review & editing. TS: Writing – original draft, Writing – review & editing.

## Footnotes

^1^https://hcv.lanl.gov/content/sequence/phyloplace/

^2^http://hcv-glue.cvr.gla.ac.uk/
