# Supplementary material for: A novel candidate hepatitis C virus genotype 4 subtype identified by next generation sequencing full-genome characterization in a patient from Saudi Arabia
Source: Front Microbiol. 2023 Nov 2;14:1285367. doi: 10.3389/fmicb.2023.1285367 (PMC10653324; doi:10.3389/fmicb.2023.1285367)
Supplement: Supplementary file 1 [file Table_1.DOCX]

**SEQUENCE**

>HCV GT 4 _141

NCCTGCTCTCTATGAGAGCAACACTCCACCATGAACCGCTCCCCTGTGAGGAACTACTGTCTTCACGCAGAAAGCGTCTAGCCATGGCGCTAGTATGAGTGTTGTACAGCCTCCAGGACCCCYCCTCCCGGGAGAGCCATAGTGGNNNNNNNNTGCTCTCTATGAGAGCAACACTCCACCATGAACCGCTCCCCTGTGAGGAACTACTGTCTTCACGCAGAAAGCGTCTAGCCATGGCGCTAGTATGAGTGTTGTACAGCCTCCAGGACCCCCCCTCCCGGGAGAGCCATAGTGGTCTGCGGAACCGGTGAGTACACCGGAATCGCCGGGATGACCGGGTCCTTTCTTGGATTTAACCCGCTCAATGCCCGGAAATTTGGGCGTGCCCCCGCAAGACTGCTAGCCGAGTAGTGTTGGGTCGCGAAAGGCCTTGTGGTACTGCCTGATAGGGTGCTTGCGAGTGCCCCGGGAGGTCTCGTAGACCGTGCACCATGAGCACGAATCCTAAACCTCAAAGAAAAACCAAACGTAACACCAACCGCCGCCCCATGGACGTTAAGTTCCCGGGTGGTGGTCAGATCGTTGGCGGAGTTTACTTGTTGCCGCGCAGGGGCCCCAGGTTGGGTGTGCGCGCGACTCGGAAGACTTCGGAGCGGTCACAACCTCGTGGAAGACGTCAGCCTATCCCCAAGGCACGTCAGTCCGAGGGCAGGTCCTGGGCCCAGCCCGGGTACCCTTGGCCCCTTTACGGCAATGAGGGCTGCGGGTGGGCAGGATGGCTCCTGTCACCCCGCGGCTCTCGGCCGTCTTGGGGCCCCAATGATCCCCGGCGGAAGTCCCGCAATTTGGGTAAGGTCATCGATACCCTGACTTGCGGCTTCGCCGATCTCATGGGATACATCCCTGTCGTAGGCGCCCCCGTGGGTGGCGTCGCCAGGGCCCTAGCGCATGGCGTCAGGGTTCTGGAGGACGGGATCAACTATGCAACAGGGAATCTCCCCGGTTGCTCCTTTTCTATCTTCCTCTTGGCACTTCTCTCGTGCTTGACTGTTCCTGCCTCAGCTGTCAACTATCGCAATGCTTCGGGCATCTATCATGTCACCAACGACTGCCCTAACTCAAGCATAATATATGAGACAGAACATCACATCTTGCACCTTCCAGGGTGTGTGCCCTGTGTCAAGGCTGAGAATGAGTCGCGGTGCTGGGTGGCTCTCACCCCCACCGTGGCAGCGCCATACGTCGGAGCTCCGCTTGAGTCTCTACGGAGGCACGTGGACCTGATGGTGGGCGCGGCCACGATGTGCTCTGCCTTCTACATCGGAGACCTGTGTGGCGGCCTCTTCCTAGTGGGTCAGATGTTCACCTTCCAACCGCGGCGCCATTGGACTACCCAGGACTGCAATTGCTCCATTTACACCGGACACATCACAGGCCACAGGATGGCCTGGGATATGATGATGAACTGGAGCCCAACGGGGACCCTGCTCCTTGCCCAACTTATGAGGATCCCATCTACTGTAGCTGATTTACTCACTGGCGGGCATTGGGGCGTCCTCGTCGGGGTGGCATATTTCAGCATGAATGCCAATTGGGCCAAAGTCATCCTGGTCTTGTTCCTTTTTGCCGGGGTCGACGCCACCACTCGCACAACTGGGGGTGTGGCAAGCGCTAACACCGCCAGTTTTGTCAGCTTGTTTTCACAAGGGTCTCAACAGAACCTGCAGCTCATTAACACCAACGGAAGCTGGCACATCAACAGGACTGCCCTCAACTGCAATGACACCTTGCACACCGGGTTTATTGCCGGTCTCCTCTATGCTCATAAGTTCAACAGCTCAGGGTGCGTCGAGCGCCTCTCAAAGTGCCGCCGTCTTGAAAGCTACTGTCAGGGCTGGGGTGCACTCAAGGCTGCTAACATTAATGAGTCGACTGGAGAAATGCCCTATTGTTGGCACTACGCACCCCGGCCATGCGGGGTGGAGCCGGCGACCACAGTGTGCGGCCCCGTGTACTGTTTCACACCTAGCCCTGTGGTGGTTGGAACAACCGACCGCCTCGGGAACCCCACCTTCAACTGGGGGGAGAATGATACTGAGGTATTCATCTTGAATTCAACTAGACCTCCAGCCGGCGGGTGGTTTGGATGCGTCTGGATGAACGGCACCGGATTCGTCAAAACCTGCGGGGCCCCCCCCTGCGCCATTACCACCAGCTGTGACAATGAGACCACCTGGAATTGTCCCACCGATTGTTTCAGGAAGCACCCGGAGACTTCATACATCAAGTGTGGTTCCGGGCCCTGGCTTACACCTCGGTGTCTGGTCCATTACCCATACCGGCTGTGGCATTTTCCCTGCACTGTCAACTACACCACATTTAAAGTCAGAATGTATGTCGGTGGTATAGAGCATCGGATGGACGTAGCATGCAACTGGACCAGGGGAGAGCCCTGCGGCCTGGAACACAGGGATCGCACTGAGCTCTCACCCCTGTTACTCTCCACTACGCAGTGGCAGGTTCTTCCCTGCTCTTTCACCACCCTGCCTGCCCTTTCGACCGGCTTGATTCACCTCCACCAGAACATCGTGGACGTCCAATACCTGTATGGCATCAGCTCCGCGGTAGTATCGTGGGCGCTCAAGTGGGAGTACGTGGTGCTCGCATTTCTGCTCCTGGCAGACGCTAGGATGTGCGCCTGCCTCTGGATGATGCTACTGGTGGCACAGGTTGAGGCGGCCCTATCCAACTTGATCACCATCAATGCTGCATCAGCCGCCGGCACACATGGCGTCGCCTGGGCCATTCTCTTCATCTGCGTGGCTTGGCATGTCAAGGGCCGGGCCCCCGCTGTCGTCACTTACGCAGCTTGCGGCATGTGGCCCCTGCTGCTCCTGCTCCTGATGTTGCCTGAAAGGGCGTACGCCTTTGACCAGGAGCTAGCAGGATCCCTCGGCGGCTGTGTTGTTGTGGCGTTAACCCTCCTAACGTTGTCCCCATATTACAAAAGATGGCTAGCGTGGGGGATGTGGTGGATACAATACCTCATTGCCAGGGCTGAGGCTCTGCTACAAGTGTATGTTCCATCCTTCAACCCGCGCGGGCCTAGGGACTCGATCATCATCCTTGCAGTCTTGGGTTGCCCATATCTCACCTTTGACATCTCCAAATATCTTTTGGCCATCCTGGGCCCAATCTACGTACTCCAGGCTTCTCTCCTACGCATCCCTTACTTCGTGAGGGCGCATGCGTTGGTTAAGATCTGCAGCTTGGTGCGTGGGGTCATGTGTGGCAAGTACTGCCAAATGGCCATGCTCAAGGTGGGAGCACTAACCGGCACTTATGTTTACAACCATCTTACCCCCCTGTCAGATTGGGCTGCCGAAGGCCTCGCTGACCTGGCCGTGGCGCTTGAACCAGTCATGTTCACGCCTATGGAGAAGAAGGTTATCACCTGGGGTGCCGACACTGCCGCATGTGGCGACATCATAAGGGGGTTGCCGGTTTCAGCTAGGCTAGGCAATGAGATCCTGCTTGGGCCGGCTGACACAGAGACGGCAAAAGGGTGGAGGCTTCTCGCCCCCATCACTGCATATGCTCAACAGACTCGGGGCTTGTTTAGCACCATCGTGACAAGCCTCACTGGCAGAGACACCAATGAGAATTCTGGCGAGGTCCAGGTCCTGTCCACCGCGACGCAGTCCTTTCTGGGCACTGCAGTCAACGGGGTCATGTGGACCGTCTACCATGGTGCGGGCTGCAAAACCATCTGCGGCCCGAAGGGACCTGTCAACCAAATGTACACCAATGTTGATCAGGATCTGGTGGGATGGCCAGCGCCGCCAGGGGTCAGGTCTCTTGCGCCGTGCACGTGCGGCTCATCGGATCTGTACCTAGTCACCCGGCACGCAGACGTGGTGCCTGTGCGGAGGCGGGGCGACAACAAGGGCGCTCTCTTGAGCCCCAGGCCTATCTCCACTCTCAAGGGGTCCTCTGGTGGTCCGCTGCTGTGCCCCATGGGGCACGCCGCTGGCATTTTCCGCGCAGCGGTGTGTACCCGAGGAGTGGCGAAAGCAGTGGACTTTGTACCAGTTGAATCCCTCGAAACCACTATGAGATCACCAGTCTTTACTGACAACTCCACGCCCCCCGCGGTGCCCCAGACCTACCAAGTCGCTCATCTGCAGGCTCCAACGGGAAGCGGAAAAAGCACCAAAGTTCCATCGGCATATGCCGCTCAAGGTTACAAAGTGCTGGTGCTTAATCCATCAGTTGCGGCTACGCTGGGTTTCGGGGCATACATGTCCAAGGCATATGGTATCGACCCTAACATCCGGACGGGCGTCAGAACCATTAATACGGGTGCGCCAATCACGTACTCGACGTATGGAAAATTTCTGGCGGATGGCGGCTGCTCTGGGGGGGCGTACGACATAATCATCTGTGACGAATGCCACTCCACTGACGCCACCACAGTCCTTGGCATAGGCACGGTCCTGGATCAAGCAGAGACCGCCGGGGTTCGCCTCGTCGTGCTTGCGACTGCTACGCCTCCGGGATCTGTGACCACCCCTCATTCCAACATAGAGGAGGTTGCTCTGCCGACGACGGGAGAAATACCTTTCTACGGCAGGGCGATCCCCCTATCGGTGGTGAAGGGGGGCAGACATCTCATCTTCTGCCATTCAAAAAAGAAATGTGACGAATTGGCCAAGCAACTGTCATCCCTTGGCCTCAACGCCGTAGCCTACTACAGGGGCTTAGACGTCTCAGTAATTCCAACATCTGGTGACGTCGTGGTGTGTGCCACGGATGCCCTTATGACTGGCTTCACCGGCGATTTTGACTCTGTAATAGACTGCAACACGTCTGTGGTACAGACTGTTGACTTCAGCTTGGACCCTACCTTCTCTATAGAGACCACCACCGTCCCCCAAGACGCGGTATCCCGCAGCCAGCGGAGAGGCCGCACCGGTAGAGGAAGGTTAGGGATATACCGGTTCGTTACTCCGGGGGAGAGACCGTCCGGCATTTTTGATACCTCAGTACTCTGCGAGTGCTATGATGCTGGATGTGCCTGGTACGAACTGACACCGGCTGAGACTACAACTAGGCTAAGGGCTTATTTCAACACACCCGGCCTCCCTGTCTGCCAGGACCACTTGGAGTTTTGGGAGAGCGTTTTCACGGGTCTTGTCAACATAGATGGCCACTTCCTGTCCCAGACTAAACAGCAAGGTGAGAACTTCCCGTACCTGGTTGCTTACCAGGCAACCGTGTGTGCCAGGGCTCTGGCCCCTCCACCAAGCTGGGACACTATGTGGAAGTGCCTAATTCGCCTTAAGCCTACTCTGCATGGGCCTACCCCTCTCCTGTATAGGTTGGGGTCCGTGCAGAATGAACTGACACTTACCCATCCTGTGACTAAATACATCATGGCCTGCATGTCGGCTGACCTCGAAGTGGTGACTAGCACGTGGGTCCTGGTGGGCGGCGTTCTGGCGGCCCTAGCTGCTTATTGTCTTTCAGTAGGCAGCGTGGTGATCGTCGGGAGGGTCGTCCTATCGGGGCAACCTGCAGTCATTCCCGACCGAGAGGTGCTCTACCGGCAGTTCGACGAGATGGAGGAGTGCTCTAAGCACCTCCCAATAGTCGAGCACGGCCTGCAACTGGCTGAGCAGTTCAAACAGAAAGCTCTCGGCGTGTTGGGTGTCGTCGGTAAGCAGGCCCAGGAAGCAACACCTGTAGTCCAGTCTCACTTCGCCAAGCTTGAGCAGTTTTGGGCAAAACATATGTGGAATTTCATCAGCGGTATCCAGTATCTTGCCGGCTTGTCTACCTTGCCCGGCAATCCTGCTATCGCATCCCTTATGTCCTTCACTGCAGCTGTCACGAGCCCCTTGTCCACCCAGCAGACCCTCCTTTTCAACATCTTAGGAGGGTGGGTGGCCTCGCAGATCGCGACCCCTACAGCCTCCACGGCATTCGTCGTGAGCGGCCTGGCGGGAGCAGCGGTCGGCAGCGTAGGCTTGGGAAAGATCCTGGTCGACATCCTCGCCGGATACGGCGCCGGCGTGGCCGGCGCCGTGGTCACTTTCAAGATCATGAGCGGCGAGATGCCTTCCACGGAAGACTTAGTCAACCTGCTTCCAGCCATTTTATCGCCGGGAGCCTTGGTGGTGGGGGTTGTGTGCGCCGCAATTCTGCGCCGCCACGTGGGGCCTGGTGAGGGGGCCGTGCAGTGGATGAACCGTCTCATTGCATTCGCATCGCGAGGCAATCACGTGTCTCCCACGCACTACGTCCCTGAAACAGACGCAGCAGCCCGCGTGACACAAATACTCACATCCCTCACCGTGACATCCCTTCTCAGACGCCTCCACAAATGGATCAATGAGGACTGTTCCACCCCGTGCGACACGTCCTGGCTGAGGGAGGTTTGGGACTGGGTCTGCACCGTGCTGAGTGACTTCAAAGTGTGGCTCAAGGCCAAGCTGATGCCCCGCTTGCCGGGTATCCCCTTTATCTCCTGCCAGAGGGGCTACAGGGGGGTATGGCGAGGCGACGGTGTGATGCACACCAGTTGCCCTTGCGGCGCAGAGCTGGCCGGCCATGTCAAGAACGGCTCGATGCGAGTCGTCGGGCCAAAGACTTGCAGTAACACCTGGCATGGGACTTTCCCCATCAACGCTTACACCACCGGTCCTAGCATACCCATCCCGGCGCCGGACTACAAGTTCGCGCTGTGGAGGGTATCTGCAGAGGAATACGTGGAGGTTCGCAGAGTGGGTGAATTCCATTACGTCACCGGGGTAACACAGGACAACCTGAAGTGCCCCTGTCAAGTTCCGGCCCCAGAGTTTTTCACGGAAGTGGACGGCGTCAGGCTACATCGTCATGCCCCCCAGTGTAAACCCATGCTGAGGGATGAGGTGTCGTTCATGGTAGGCCTCAACACCTTCGTGGTGGGGTCTCAGCTTCCATGTGAGCCGGAGCCGGACGTGGCTGTGTTGACGTCCATGCTGACAGATCCATCACATATAACGGCGGAAACGGCGGGCCGTAGACTAAAGCGGGGGTCCCCACCCTCGCTAGCCAGTTCCTCGGCTAGCCAGTTGTCCGCCCCGTCCCTCAAAGCCACATGCACTGACGACCACAACGCCCCGGGTATAAACCTCATTGAGGCCAACCTCTCATGGGGTACCAAAGTCACCCAGGTCGAGACGGACGACAAGGTGGTAGTGCTAGATACTTTTGAACCACTCGTGGCAGAACATGATGACAGGGAAGTCTCCGTCGCAGCCGAGATCTTGCTGCCTTCCAAGAAATTCCCTCCAGCTTTGCCGCTGTGGGCCCAGCCGAGCTATAACCCGCCCCTGGTTGAGGCGTGGAAGCGGCCAGACTACGACCCCCCGGTCGTCCATGGCTGCGCGCTGCCTCCCAGCAAGCAAGCTCCCGTTCCTCCACCCAGGAGGAAGCGGGCAATACAGCTCACTGAGTCCACCGTCTCCGAGGCACTGGCAGAGCTGGCTGTCAAGACCTTCGGGCAGTCGACGCCCGATTCGGACTCTGGCGTTGACCCCATTGCCCCAACTGAACCTTCAACTTCGGCCCCCTCAACCATCGACGACATGTCCGACAACGAATCTTATTCGTCGATGCCTCCATTGGAGGGAGACGCCGGTGACCCAGATTTGTCCTCCGTTGCCGGTGAAGACGAGGTAGTATGCTGCTCAATGTCCTATTCATGGACAGGGGCGCTTGTGACACCTAGTGCAGCAGAAGAGTCAAAGCTGCCAATTAGCCCCCTGAGCAACTCACTGCTACGCCACCACAATATGGTGTATGCCACGACCTCTCGTTCGGCCGTTACCCGGCAGAAGAAGGTCACTTTCGACCGCATGCAAGTGGTGGACAGTCATTACCATGAGGTACTCAAGGAGATCAAGACACGAGCGTCCAAAGTGAAGGCACGCTTGCTCACTACAGAGGAAGCATGTGACCTGACGCCCCCTCATTCAGCCAAATCCAAGTTCGGCTACGGGGCAAAGGATGTTCGGAGTCATGCCCGCAAGGCCGTTAACCACATCAACTCCGTGTGGGAGGACTTGCTGGAGGACAACAACACCCCTATTCCGACAACAATCATGGCTAAAAACGAAGTTTTCTGCGTAAAGCCAGAAAAAGGCGGCCGAAAGCCTGCCCGTCTGATCGTATACCCGGACCTAGGAGTGCGGGTCTGCGAGAAGAAGGCGCTTTATGACGTCGTCAAACAACTTCCTGAAGCCGTGATGGGAGCCGCTTACGGCTTCCAATACTCCCCAGCGCAGCGGGTCGATTACCTCTTAGCCGCTTGGGGATCCAAACGGGTCCCCATGGGGTTCTCCTATGACACCCGCTGCTTTGATTCCACTGTGACTGAGAGGGACATCAGGGTCGAAGAAGAAGTCTATCAGTGTTGTGACCTGGAGCCTGAGGCCCGCAAGGTAATTACAGCCCTCACAGAAAGACTCTATGTGGGCGGCCCCATGCATAATAGCAGGGGAGACCTATGCGGACTCCGCAGGTGCCGCGCGAGCGGTGTCTTCACCACCAGCTTCGGAAACACACTGACGTGCTACCTTAAAGCCTCCGCCGCCATCAAGGCGGCCGGCATAAAGGACAGCACCATGTTGGTATGCGGTGACGACTTAGTCGTCGTCGCTGAGAGCGGCGGCGTGGAGGAGGACAAGAGAGCCCTCGGAGCCTTCACGGAGGCTATGACGAGGTACTCAGCTCCCCCCGGAGACGCGCCGCAGCCAGCATATGACTTGGAGCAAATAACATCATGCTCATCCAACGTCTCAGTCGCACACGATGCGACGGGCAAGAGGGTATATTACTTGACCCGAGACCCTGAGACCCCCCTGGCGCGAGCCGCCTGGGAAACAGTCCGACACACTCCAGTAAACTCCTGGCTGGGTAACATCATAATCTACGCGCCCACGATATGGGTGCGAATGGTGCTGATGACCCATTTCTTTTCAATACTCCAAAGCCAGGAAGCCCTTGAAAAAGCACTGGACTTCGACATGTACGGAGTCACATACTCCATCACTCCGCTGGATTTACCAGCCATCATCCAAAGACTCCACGGCTTGAGCGCATTTACGCTGCACGGATACTCTCCACACGAACTTAACCGGGTGGCAGGAACCCTCAGAAAACTTGGGGTACCCCCGTTGAGAGCGTGGAGACATCGGGCCCGAGCAGTTCGCGCCAAGCTCATCGCCCAGGGAGGGAAAGCCAAAATCTGTGGCGTGTACCTCTTCAACTGGGCGGTGAAGACCAAACTCAGACTCACTCCATTGTCCGCCGCGGCCAAGCTCGACTTGTCGGGCTGGTTCAAGGTGGGCGCCGGCGGGGGAGACATTTATCACAGCGTGTCTCATGCCCGACCCCGCTACTTACTCCTGTGCCTACTCCTACTTTCCGTAGGGGTAGGCATCTTTCTGCTGCCTGCTCGATAGGCAGCTTGACACTCCGACCTBAG
